# Dietary intake of cod and scallop reduces atherosclerotic burden in female apolipoprotein E-deficient mice fed a Western-type high fat diet for 13 weeks

**DOI:** 10.1186/s12986-016-0068-z

**Published:** 2016-02-02

**Authors:** Ida-Johanne Jensen, Mari Walquist, Bjørn Liaset, Edel O. Elvevoll, Karl-Erik Eilertsen

**Affiliations:** Norwegian College of Fishery Science, Faculty of Biosciences, Fisheries and Economics, UIT - The Arctic University of Norway, N-9037 Tromsø, Norway; National Institute of Nutrition and Seafood Research, 5004 Bergen, Norway

**Keywords:** Apolipoprotein E-deficient mice, Atherosclerosis, Plaque burden, Dietary intake, cod, Scallop, Lean seafood, Gene expression

## Abstract

**Background:**

It is now increasingly recognized that the beneficial effects of seafood consumption is not limited to lipids and fatty acid, but that the protein part, i.e., peptides and amino acids, together with vitamins and even unknown bioactive constituents also are important for disease prevention. This study was designed to evaluate the putative anti-atherogenic effects of different protein sources (a lean seafood and a nonseafood) in apolipoprotein E-deficient (apoE^−/−^) mice.

**Methods:**

Twenty-four 5-week-old female apoE^−/−^ mice were fed Western type diets containing chicken or a combination of cod and scallops as dietary protein sources for 13 weeks. Atherosclerotic plaque burden, weight, serum levels of leptin, glucose and LDL cholesterol as well as gene expressions from liver and heart were evaluated. Statistical analyses were performed using SPSS. Differences between the variables were evaluated using independent *t*-test or Mann–Whitney *U* test for normally and non-normally distributed variables, respectively. Normality was defined by the Shapiro-Wilk test.

**Results:**

The mice fed cod-scallop had a 24 % (*p* < 0.05) reduced total aorta atherosclerotic plaque burden compared to the chicken fed group, whereas the reduction in plaque in the less lesion prone thoracic and abdominal parts of the descending aorta were 46 % (*p* < 0.05) and 56 % (*p* < 0.05), respectively. In addition, mice fed cod-scallop gained less weight, and had lower serum levels of leptin, glucose and LDL cholesterol, compared to those fed chicken. Analysis of expression of the genes from liver and heart showed that hepatic endogenous antioxidant paraoxonase 2 (*Pon2* gene) and the vascular cell adhesion molecule VCAM-1 (*Vcam1* gene) were down regulated in mice fed cod-scallop compared to mice fed chicken.

**Conclusion:**

The present study revealed a metabolic beneficial effect of lean seafood compared to chicken, as atherosclerotic plaque burden, serum glucose, leptin and LDL cholesterol levels were reduced in mice fed cod-scallop.

**Electronic supplementary material:**

The online version of this article (doi:10.1186/s12986-016-0068-z) contains supplementary material, which is available to authorized users.

## Background

In spite of tremendous developments in preventive and acute treatment of cardiovascular diseases (CVD), CVD is still the largest cause of morbidity and pre-mature mortality worldwide [1]. Important factors that contribute to this is the increased consumption of energy dense and processed foods together with a decline in physical activity [2]. Atherosclerosis is a multifactorial inflammatory condition affecting the arteries by plaque formation and subsequent narrowing of the lumen. Diet has a major impact on the atherosclerotic disease initiation and progression and nutritional intervention is considered an effective and safe approach to health maintenance and CVD prevention [3]. Indeed, a change in nutritional pattern has been estimated to reduce CVD-related deaths by up to 60 % [4].

A diet high in fish and seafood has long been acknowledged to reduce risk of CVD [5–7], mainly due to the effects of the omega-3 fatty acids EPA (eicosapentaenoic acid, 20:5n3) and DHA (docosahexaenoic acid, 22:6n3). It has, however, become increasingly evident that the beneficial effects of seafood consumption are not limited to lipids and fatty acids. Proteins, peptides and amino acids together with vitamins and even unknown bioactive constituents may also be important for disease prevention by controlling circulating levels of cholesterol, lipoproteins and triglycerides, increasing endogenous antioxidants and lowering blood pressure [8–13]. The exact mechanisms behind these effects, however, remain unknown.

Seafood is generally recognized as a rich source of taurine [14] which might have a positive impact on CVD [15, 16]. Recently, it was shown that male C57BL/6J mice fed a high-fat, high-sugar diet containing scallop, endogenously high in taurine and glycine, as protein source, had improved plasma lipid profile, as compared to mice fed high fat, high sugar diets with chicken fillet or cod as the protein sources [17]. Thus, the present study was undertaken to elucidate whether intake of a Western-type diet with a mixture of cod and scallop protein, as compared to chicken fillet, would prevent development of CVD in the atherosclerosis-prone model Apolipoprotein E-deficient (apoE^−/−^) mice.

A substantial metabolic beneficial effect of the marine protein sources was documented, as atherosclerotic plaque burden, serum glucose, leptin and LDL cholesterol levels were reduced in mice fed cod-scallop.

## Methods

### Diets

The mice were fed a Western type diet (Research diet D12079B, 17 energy percent protein, 43 energy percent carbohydrates and 40 energy percent fat, Table 1) in which the standard protein source casein was fully exchanged with either chicken fillet or a mixture of cod fillet and scallop muscle (1:1 on nitrogen basis). The amino acid methionine was added to ensure adequate metabolic balance. The lipid, protein and energy contents were 20–21g/100g, 15.9–16.9g/100g and 20.2–21.0kJ/g, respectively, and the cholesterol contents were 2.0 and 1.9g/kg for the chicken and cod-scallop diet (Table 2). The amino acid compositions were similar between the two diets (Table 3) with a few exceptions. Histidine was more abundant in chicken than in the cod-scallop diet, while the cod-scallop diet had almost twice the amount of glycine compared to chicken diet (10.1 and 5.7mg/g, respectively). Further, the cod-scallop diet had a taurine content 5.3mg/g, whereas chicken diet was almost devoid of taurine. The percentage distribution of fatty acids (Table 4) where similar between the diets with the exceptions of EPA and DHA which were higher in cod-scallop diet compared to chicken diet and arachidonic acid which was higher in the chicken diet compared to the cod-scallop diet.

**Table 1:** Composition of experimental diets (g/kg)

	Dietary treatments	
	Chicken	Cod-scallop
Chicken fillet	189.8	0
Cod fillet	0	92.9
Scallop	0	102.9
L-methionine	3	3
Corn starch	55.6	49.7
Maltodextrine	100	100
Sucrose	341	341
Cellulose	50	50
Milk fat (anhydrous)	200	200
Corn oil	10	10
Mineral mix S1001	35	35
Calcium carbonate	4	4
Vitamin mix V1001	10	10
Choline bitartrate	2	2
Cholesterol	1.1	1.1
Ethoxyquin	0.04	0.04

**Table 2:** Analyzed composition of the experimental diets (g/kg)

	Dietary treatments	
	Chicken	Cod-scallop
Crude protein (N*6.25)	169	159
Fat	210	200
Cholesterol	2.0	1.9
Gross energy (kJ/g)	21	20.2

**Table 3:** Amino acid composition of the experimental diets (mg/g)

	Dietary treatments	
	Chicken	Cod-scallop
Hydroxyproline	0.2	0.2
Histidine	4.5	2.2
Taurine	0.1	5.3
Serine	5.8	5.7
Arginine	8.1	8.9
Glycine	5.7	10.1
Asparagine + aspartic acid	15.4	15.2
Glutamine + glutamic acid	23.6	22.2
Threonine	6.6	5.7
Alanine	9.0	8.3
Proline	5.2	4.1
Lysine	14.7	13.2
Tyrosine	3.5	3.0
Methionine	6.3	6.3
Valine	7.6	6.1
Isoleucine	7.3	6.1
Leucine	12.0	10.6
Phenylalanine	5.5	4.7
Cysteine	1.9	2.1
Tryptophan	1.62	1.23
SAA	144.52	135.93
EAA^1^	66.12	56.13
BCAA^2^	26.9	22.8

*SAA* sum amino acids; ^1^Sum of essential amino acids (histidine, isoleucine, leucine, methionine, lysine, valine, tryptophane, threonine, phenylalanine). ^2^Sum of branched-chain amino acids (isoleucine, leucine and valine)

**Table 4:** Fatty acid (FA) composition as percentage of total FA and mg/g of the experimental diets

	Dietary treatments			
	Chicken		Cod-scallop	
FA	%	mg/g	%	mg/g
<14:00	8.4	15.7	8.2	14.3
14:00	10.3	19.1	10.6	18.4
16:00	28	52.0	28.2	48.8
16:1n7	1.5	2.83	1.5	2.56
18:00	10.7	20.0	10.6	18.4
18:1n9	23.6	43.9	22.6	39.1
18:1n7	1.0	1.8	1	1.7
18:2n6	5.4	10.0	4.2	7.27
18:3n3	0.7	1.2	0.6	0.97
20:1n9	0.1	0.2	0.1	0.17
20:4n6	0.2	0.4	0.1	0.24
20:5n3	0.1	0.1	0.5	0.79
22:5n3	0.1	0.23	0.1	0.21
22:6n3	0.1	0.17	0.7	1.25
SFA	59.2	110	59.6	103
MUFA	29.7	55.2	28.8	49.9
n-3	1.0	1.76	1.9	3.22
n-6	5.6	10.4	4.3	7.51
n6/n3	5.6	5.9	2.3	2.3
Sum identified	96.1	179	95.3	165
Sum nonidentified	3.9	7.29	4.7	8.08

### Protein, lipid and energy content of feed

The feed was analyzed for crude protein, total fat and gross energy content. Nitrogen content was determined after combustion using the Dumas method (Leco FP-528, ISO 34/SC 5, ON, Canada) and crude protein content was calculated as nitrogen content multiplied by 6.25. Gross energy was determined using an automatic adiabatic calorimeter (Model 1241; Parr Instruments, Moline, IL, USA). Total fat was determined gravimetrically by extraction with organic solvents before and after acidic hydrolysis, as described previously [17]. The amino acid composition was determined after acidic hydrolysis by the Acquity UPLC system (Waters, USA), the tryptophan content was determined after basic hydrolysis on a HPLC system (Shimadzu, USA) as described previously in details [17]. Total cysteine in the samples was determined after oxidation of cysteine/cystine with 9:1 performic acid (88 %): H_2_O_2_ (30 %) (v/v) to yield cysteic acid. Total cysteine analysis was performed by the Norwegian Institute of Food, Fishery and Aquaculture (Bergen, Norway). Total cholesterol was determined on a Thermo Trace 2000 GC equipped with a flame ionization detector (FID) operated under conditions described elsewhere [18]. Prior to injection on the GLC, the samples had been added internal standard α-5 cholestan, saponified in 0.5M NaOH/MeOH at 80 °C for 15 min, hexan-extracted, dried under N_2_ at 60 °C, and resuspended in hexan.

### Animals and housing

Apolipoprotein E-deficient mice fed a Western type, high fat diet, were used as model system. Such apoE^−/−^ mice develop atherosclerosis spontaneously with similar characteristics as those seen in humans [19, 20] and are extensively used to study dietary effects on atherosclerosis. Twenty-four female apoE^−/−^ mice (B6.129P2-*Apoe*^*tm1Unc*^N11), 5-weeks-old, were obtained from Taconic, Denmark. After one week of acclimation, the mice were ear marked and randomly assigned to the experimental groups (*n* = 12/group) with equal number of cages per treatment (*n* = 4 cages/diet). All mice were housed in individually ventilated cages in the same room at 21 °C and 55 % relative humidity, on a 12 h day/night cycle (light: 0600 to 1800 h) in a conventional laboratory animal unit. The mice had unrestricted access to feed that was distributed in wire bar lids with food hoppers for 13 weeks. Cages and bedding were changed once per week. After 13 weeks of feeding, the mice were feed-deprived for 3 h before they were euthanized by carbon dioxide inhalation. Blood was drawn by cardiac puncture and serum separated and frozen at −80 °C. Tissues were dissected out, weighted, snap frozen and kept at −80 °C. The study was approved by the Norwegian Animal Research Authority (Approval number 3277) and performed following Federation of European Laboratory Animal Science Associations recommendations and according to the Norwegian legislation on the care and use of experimental animals. The mice were pathogen free, stated by a health certificate. Adverse events were not observed.

### Analysis of atherosclerosis

After blood removal by cardiac puncture, the mouse was immediately perfused through the left ventricle with sterile saline (0.9 %) for approximately 5 min until no residual blood was apparent in the circulation. The entire aorta from the proximal ascending aorta to the bifurcation of the iliac arteries was dissected and cleaned in situ from periadventitial fat. The aorta was opened longitudinally, fixed and prepared by lipid Oil Red O-staining and subjected to whole mount *en face* evaluation as previously described [21]. Aorta images were made by scanning the objective on a high resolution scanner and lesion areas were evaluated using ImageJ software [22]. The extent of atherosclerotic burden was reported as percentage of the total area of an artery, or artery region, i.e., the aortic arch, the thoracoabdominal part of the descending aorta (thoracic area), and the infrarenal part down to the iliac bifurcation (abdominal area), occupied by atherosclerotic lesions.

### RNA-isolation

Total RNA was isolated from liver and heart tissue. Immediately after perfusion, tissue was dried, weighted and frozen in liquid nitrogen and kept at −80 °C until analysis. Tissue (30mg to 90mg) was cut in liquid nitrogen and homogenized in 1 ml Trizol (Invitrogen) using a bead miller (Precellys 24, Bertin Technologies). After centrifuging the homogenate for 10 min at 12000g and 4 °C, the supernatant was mixed generously with 0.2 volumes chloroform, incubated 30 min on ice and centrifuged for 20 min at 12000g and 4 °C. The aqueous phase was mixed with 0.5 volumes fresh isopropanol and incubated on ice for a minimum of 2 h before 30 min centrifugation at full speed (21000g) and 4 °C. The remaining pellet was washed with ice-cold 80 % ethanol, centrifuged 5 min at 21000g and 4 °C, dried and dissolved in RNA storage solution (Ambion) and stored at −80 °C. RNA concentration was determined using a Qubit 1.0 fluorometer (Life technologies) and the RNA integrity was evaluated using a 2100 Bioanalyzer Instrument (Agilent Technologies). The relative integrity numbers of the isolates were between 8.1 and 9.4.

### Reverse transcription and quantitative real-time PCR

Reverse transcription was performed in triplicate using the high capacity cDNA RT kit (Applied Biosystems) with 50ng RNA in 20μl reactions. Four μl cDNA (12.5ng of reverse transcribed RNA) per 20μl reaction was analyzed by quantitative RT-PCR using predesigned TaqMan® Gene Expression assays (Additional file 1: Table S1) and TaqMan Fast Universal PCR Master Mix (Applied Biosystems) in 96-well plates with an ABI Prism 7500 Fast Cycler (Applied Biosystems). The amplification profile was 95 °C for 20s followed by 40 cycles of 95 °C for 3s and 60 °C for 30s. Endogenous reference genes were selected using the TaqMan Array Mouse Endogenous Control assays. From this panel of 32 housekeeping genes, the two most stably expressed control genes (*Hprt* and *Tbp*) were selected, and the geometric mean of the two was used to normalize gene expressions. The calculations were made using the relative expression software tool [23]. All assays included no-template-controls and an inter plate calibrator.

### Serum cholesterol, triglyceride, glucose and total protein concentrations

The serum cholesterol, LDL cholesterol, triglyceride, glucose and total protein concentrations were determined by conventional enzymatic kits using a MaxMat bioanalyzer (MaxMat PL II, Montpellier, France).

### Hepatic fat and fatty acid analysis

Total hepatic fat was extracted using dichloromethane and methanol, and determined gravimetrically [24]. The fatty acid composition of the liver samples was determined as previously described [25].

### Serum cytokines, chemokines and hormones

Serum samples were analyzed in duplicates according to the manufacturers’ instructions using a MSD Mouse Proinflammatory panel 1 V-Plex kit (MULTI-ARRAY®, Meso Scale Discovery, Gaithersburg, MD) and a Bio-Plex Pro Mouse Diabetic Assay (MAGPIX, Bio-Rad, Copenhagen, Denmark). Serum cytokines, chemokines and hormones analyzed were IFN-γ, IL-1β, IL-2, IL-4, IL-5, IL-6, IL-10, IL-12p70, KC/GRO, and TNF-α using MSD and glucagon, insulin, leptin, monocyte chemotactic protein-1 (MCP-1), and regulated upon activation, normal T cell expressed and secreted (RANTES) using Bio-Plex (Bio-Rad).

### Statistical analysis

The results are presented as mean ± standard error of the mean. SPSS 19.0 (SPSS Inc., Chicago, IL) was used to perform statistical analysis of the data. The Shapiro-Wilk test was performed to determine the distribution of the variables and non-normal distributions were log-transformed before statistical analysis with an independent *t*-Test. Variables that were non-normally distributed after log-transformation were analyzed with Mann–Whitney *U* test. Bivariate analyses of correlation between parameters were assessed by Pearson’s correlation coefficient. Differences were considered significant when *p* < 0.05.

## Results

### General outcomes and mice growth

**Fig. 1:**
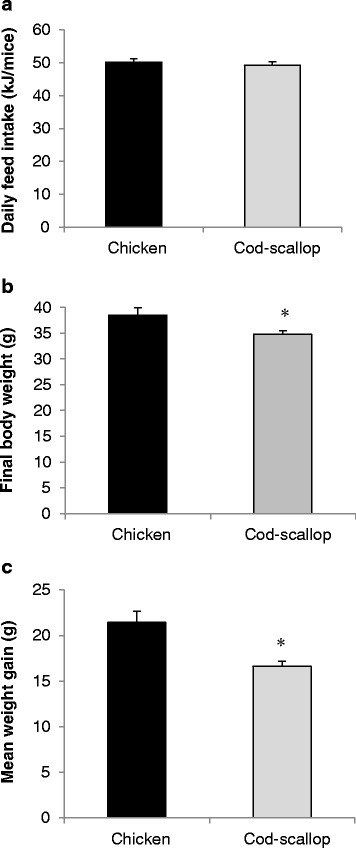
Feed intake and weight. (**a**) average daily feed intake (kJ/mice); (**b**) final body weight; (**c**) body weight gain (g) of female apolipoprotein E-deficient mice fed diets containing different protein sources for 13 weeks. Data are mean ± SEM. Average daily feed intake was analyzed using the non-parametric Mann–Whitney *U* test, while final body weight and body weight gain were analyzed using the independent samples *t*-Test. * denotes significant difference between mice fed chicken protein (*n* = 12) and mice fed cod-scallop protein (*n* = 10)

**Fig. 2:**
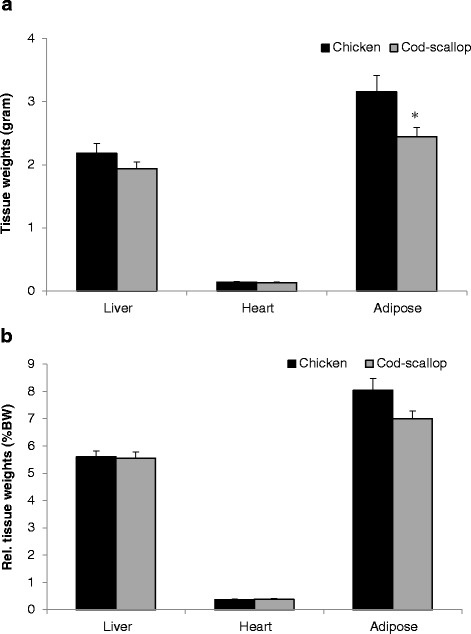
Tissue weights and relative tissue weights of female apolipoprotein E-deficient mice. The mice were fed diets containing different protein sources for 13 weeks. Data are mean ± SEM. **a** Data from liver and visceral adipose tissue were analyzed using the independent samples *t*-Test. Data from heart were analyzed using the non-parametric Mann–Whitney *U* test. * denotes significant difference in tissue weight between mice fed chicken protein (*n* = 12) and mice fed cod-scallop protein (*n* = 10). **b** Data from liver were analyzed using the independent samples *t*-Test. Data from adipose tissue were log transformed before analysis using the independent samples *t*-Test. Data from heart were analyzed using the non-parametric Mann–Whitney *U* test. * denotes significant difference in relative tissue weight between mice fed chicken protein (*n* = 12) and mice fed cod-scallop protein (*n* = 10)

The mice appeared in good physical shape and gained weight throughout the experimental period. The exceptions were two mice in the cod-scallop group that were euthanized due to weight loss during the first weeks of the study. These two mice were small at arrival and should probably have been removed prior to the start of the study. Although the average daily energy intake was similar in both groups (*p* = 0.88), the mice fed chicken protein had gained significantly more weight than mice fed cod-scallop protein (21.4g vs 16.6g, *p* < 0.05) at the end of the experiment (Fig. 1). The visceral adipose tissue, represented by the epididymal and the perirenal fat, weight was higher (3.15g vs 2.45g, *p* < 0.05) in the chicken fed group compared to the cod-scallop fed group (Fig. 2a), but when calculated as relative to body weight, no significant difference was observed (Fig. 2b). Heart weight was not different between cod-scallop and chicken fed mice.

### Atherosclerotic lesions

**Fig. 3:**
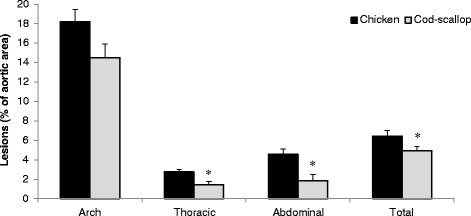
Atherosclerotic plaque burden. The plaque burden is expressed as area percentage covered by lipid Oil Red O staining, in female apolipoprotein E-deficient mice fed diets containing different protein sources for 13 weeks. Data are mean ± SEM and were analyzed using the independent samples *t*-test. * denotes significant difference in atherosclerotic lesion between mice fed chicken protein (*n* = 12) and mice fed cod-scallop protein (*n* = 10)

The atherosclerotic lesions were predominantly distributed in the aortic arch and in the areas surrounding the branching points of the major arteries (Fig. 3). The plaque burdens in the aorta thoracic, abdominal as well as total area were reduced by 46 %, 56 % and 24 % (*p* = 0.004, 0.003 and 0.044), respectively in mice fed cod-scallop compared to mice fed chicken. Also in the most plaque prone area, the aortic arch, the cod-scallop fed mice had lower plaque burden, however, the difference did not reach statistical significance (*p* = 0.09).

### Hepatic fat content and fatty acid composition

The hepatic fat content extracted with dichloromethane/methanol was higher in chicken fed mice compared to cod-scallop fed mice (430mg/g and 330mg/g, respectively, *p* < 0.05) (Table 5). The concentration of hepatic fatty acids was borderline (*p* = 0.054) higher in chicken fed mice compared to cod-scallop fed mice, 194 versus 152mg/g liver, respectively. When estimating total liver fatty acid amount (fatty acid concentration x liver mass), the chicken fed mice had elevated amounts of total hepatic fatty acids (Table 5). The fatty acid compositions evaluated in liver tissues (Table 6) showed that the group receiving the cod-scallop diet had higher (*p* < 0.001) percentage of saturated fatty acids (SFA) compared to the chicken group (30 % and 25 %, respectively). The percentage of linoleic acid was similar (approximately 5.0 %), EPA was only detected in the cod-scallop group, and both DPA and DHA were higher (*p* < 0.001) in the cod-scallop fed group compared to the chicken fed group (0.7 and 0.5 % DPA and 3.7, and 1.2 %DHA, respectively). However, when presented as mg/g liver tissue, the group receiving the cod-scallop diet had similar levels of SFA, lower levels of MUFA, and higher levels of EPA, DPA and DHA. The n6/n3 ratio was higher in the chicken fed group (4.3 vs 1.2) and the opposite was true for SFA/monounsaturated fatty acids (MUFA) ratio (0.4 in the chicken fed group and 0.6 in the cod-scallop fed group). The 16:1n7/16:0 ratios were 0.20 and 0.15 for chicken fed and cod-scallop fed mice, respectively.

**Table 5:** Lipid content and total fatty acids (FA) (mg/g) of hepatic tissue from apoE^−/−^ mice

	Dietary treatments	
	Chicken (*n* = 12)	Cod-scallop (*n* = 10)
Lipid (mg/g liver)	430 ± 23	330 ± 35*
FA(mg/g fat)	194 ± 11	153 ± 16
FA (mg/g)*liver weight	423 ± 37	320 ± 28^*^

The mice were fed different protein sources for 13 weeks. Data are mean ± SEM (*n* = 10–12). Data were analyzed using the independent samples *t*-Test. *indicate significant difference (*p* < 0.05) between diet groups

**Table 6:** Fatty acid (FA) composition as percentage of total FA and mg/g of hepatic tissue from apoE^−/−^ mice

	Dietary treatments			
	Chicken (*n* = 12)		Cod-scallop (*n* = 10)	
FA	%	mg/g	%	mg/g
14:00	0.98 ± 0.0	1.88 ± 0.1	0.87 ± 0.0	1.34 ± 0.2*
16:00	22.5 ± 0.3	43.6 ± 2.6	25.09 ± 0.6*	38.51 ± 4.0
16:1n7	4.61 ± 0.4	8.96 ± 0.9	3.74 ± 0.2	5.66 ± 0.5*
18:00	3.08 ± 0.1	5.91 ± 0.3	4.13 ± 0.2*	6.17 ± 0.5
18:1n9	45.03 ± 1.4	87.6 ± 6.0	41.95 ± 0.7*	64.44 ± 6.6*
18:1n7	5.12 ± 0.4	9.92 ± 0.9	3.55 ± 0.1*	5.38 ± 0.5*
18:2n6	4.93 ± 0.3	9.59 ± 0.8	4.96 ± 0.1	7.55 ± 0.7
20:1n9	0.63 ± 0.0	1.24 ± 0.1	0.42 ± 0.0*	0.60 ± 0.1*
20:4n6	1.25 ± 0.1	2.42 ± 0.2	1.09 ± 0.1	1.57 ± 0.1*
20:5n3	Bdl		0.95 ± 0.1	1.40 ± 0.1
22:5n3	0.52 ± 0.1	0.98 ± 0.1	0.71 ± 0.1*	1.04 ± 0.1*
22:6n3	1.18 ± 0.1	2.28 ± 0.2	3.66 ± 0.2*	5.38 ± 0.3*
SFA	24.63 ± 2.0	47.58 ± 4.7	30.09 ± 0.6*	46.02 ± 4.6
MUFA	55.40 ± 2.1	107.68 ± 7.7	49.54 ± 0.8*	75.92 ± 7.6*
n-3	1.70 ± 0.1	2.97 ± 0.3	5.31 ± 0.3*	7.82 ± 0.5*
n-6	6.18 ± 0.4	12.01 ± 1.0	6.05 ± 0.2	9.12 ± 0.8*
n6/n3	4.27 ± 0.3		1.2 ± 0.0*	
16:1n7/16:0	0.20		0.15	
Sum identified	87.78 ± 0.7	170.30 ± 10.3	91.04 ± 0.7	138.93 ± 4.7
Sum unidentified	12.22 ± 0.7	23.35 ± 1.4	8.97 ± 0.7	13.52 ± 2.1

The mice were fed diets containing different protein sources for 13 weeks. Data are mean ± SEM (*n* = 10–12). Data were analyzed using the non-parametric Mann–Whitney *U* test. *indicate significant difference (*p* < 0.05). Bdl, below detection limit of 0.5 area %

### Serum cholesterol and lipid levels

Serum LDL-cholesterol was significantly lower in the cod-scallop fed mice compared to the chicken fed mice, 12.5 vs 14.0 mmol/l (*P* < 0.05), however, neither serum concentrations of total cholesterol nor triglyceride differed significantly between the chicken and cod-scallop fed groups (Table 7).

**Table 7:** Serum lipids, glucose and total protein concentrations in apoE^−/−^ mice

	Dietary treatments	
	Chicken (*n* = 12)	Cod-scallop (*n* = 10)
Cholesterol (mmol/l)	24.20 ± 0.9	21.84 ± 0.7
LDL-Cholesterol (mmol/l)	13.97 ± 0.57	12.49 ± 0.37*
Triglycerides (mmol/l)	1.90 ± 0.2	2.16 ± 0.2
Glucose (mmol/l)	19.60 ± 1.0	16.68 ± 0.6*
Total protein (g/l)	95.40 ± 3.7	86.76 ± 4.5

The mice were fed diets containing different protein sources for 13 weeks. Data are mean ± SEM (*n* = 10–12). Data were analyzed with the independent samples *t*-Test. Data for triglycerides were analyzed using the non-parametric Mann–Whitney *U* test. *indicate significant difference (*p* < 0.05) between dietary treatments

### Serum glucose, protein, cytokine, chemokine and hormone concentrations

Serum glucose concentrations differed between mice fed cod-scallop and chicken, being lower (*p* < 0.05) in the cod-scallop fed group (16.7mmol/l) compared to the chicken fed group (19.6mmol/l) (Table 7). Total protein content in serum did not differ between cod-scallop and chicken fed mice (95.4g/l and 86.8g/l, respectively) (Table 7). Leptin was higher (*p* < 0.01) in the chicken fed group (40.3pg/ml) compared to the cod-scallop fed group (25.4pg/ml) (Table 8). Serum levels of the cytokines and chemokines IFN-γ, IL-1β, IL-2, IL-4, IL-5, IL-6, IL-10, IL-12p70, KC/GRO, TNF-α, MCP-1, and RANTES did not differ between the groups (data now shown). Three hours feed-deprived serum concentrations of insulin and glucagon did not differ between the two groups (Table 8). Insulin concentrations were 1.4ng/ml in both groups and glucagon concentrations were 700pg/ml and 1000pg/ml for chicken and cod-scallop fed mice, respectively.

**Table 8:** Serum concentrations of glucagon, insulin and leptin in apoE^−/−^ mice

	Dietary treatments	
	Chicken (*n* = 12)	Cod-scallop (*n* = 10)
Glucagon (pg/ml)	698 ± 202	1054 ± 333
Insulin (ng/ml)	1.4 ± 0.4	1.4 ± 0.5
Leptin (pg/ml)	40.3 ± 11.6	25.4 ± 8.0*

The mice were fed diets containing different protein sources for 13 weeks. Data are mean ± SEM (*n* = 10–12). Data for leptin were analyzed using the independent samples *t*-Test. Data for insulin were log transformed before analysis using the independent samples *t*-Test. Data for glucagon were analyzed using the non-parametric Mann–Whitney *U* test. * indicate significant difference (*p* < 0.05) between dietary treatments

### Gene expression

The gene expression assays used for analysis of liver were chosen to include genes involved in cholesterol, lipoprotein and lipid metabolism, inflammatory response and endogenous antioxidant defense, possibly involved in several phases of the atherosclerotic process (Table 9). Hepatic expression of the genes encoding the antioxidative protein paraoxonase 2 (*Pon2*) and vascular cell adhesion protein 1 (*Vcam1*) were down regulated in mice fed cod-scallop compared to mice fed chicken. Hepatic expression of all the other investigated genes were unaffected by the dietary intervention. The effects of the two diets on the cardiac function and homeostasis were also investigated by means of gene expression analysis of a battery of genes related to heart function, fibrosis and apoptosis. There were, however, no significant differences between cardiac gene expression of mice fed chicken or cod-scallop (Table 9).

**Table 9:** Relative gene expressions in liver and heart of apoE^−/−^ mice

Gene	Chicken	Cod-scallop	Gene	Chicken	Cod-scallop
*Liver*			Heart		
*Cytokines and chemokines*			*Cytokines and chemokines*		
Ccl2	*1.0 ± 0.11*	*0.85 ± 0.05*	Ccl2	*1.0 ± 0.12*	*0.80 ± 0.10*
Il1b	*1.0 ± 0.13*	*0.86 ± 0.05*	Ilb1	*1.0 ± 0.16*	*1.20 ± 0.38*
Icam1	*1.0 ± 0.13*	*0.97 ± 0.05*	Icam1	*1.0 ± 0.06*	*1.03 ± 0.11*
Vcam1	*1.0 ± 0.06*	0.81 ± 0.06*	*Cardiac function and homeostasis*		
*Endogenous antioxidant system*			Agtr1	*1.0 ± 0.08*	*1.09 ± 0.13*
Pon2	*1.0 ± 0.04*	0.84 ± 0.04*	Ankrd1	*1.0 ± 0.13*	*1.03 ± 0.24*
Nfe212	*1.0 ± 0.05*	*0.94 ± 0.04*	Nppa	*1.0 ± 0.31*	*1.17 ± 0.43*
Sod1	*1.0 ± 0.05*	*1.04 ± 0.07*	Nppb	*1.0 ± 0.21*	*1.07 ± 0.34*
Sod2	*1.0 ± 0.03*	*1.05 ± 0.03*	Myh6	*1.0 ± 0.04*	*1.10 ± 0.08*
Gpx1	*1.0 ± 0.05*	*1.01 ± 0.04*	Myh7	*1.0 ± 0.15*	*0.80 ± 0.12*
Gpx4	*1.0 ± 0.04*	*1.04 ± 0.04*	*Angiogenesis*		
Ucp2	*1.0 ± 0.13*	*0.94 ± 0.08*	Vegfb	*1.0 ± 0.06*	*1.07 ± 0.15*
*Cholesterol and lipoprotein metabolism*			*Apoptosis*		
Abcg5	*1.0 ± 0.26*	*0.89 ± 0.09*	Bcl2	*1.0 ± 0.07*	*0.99 ± 0.07*
Abcg8	*1.0 ± 0.25*	*0.77 ± 0.07*	Casp3	*1.0 ± 0.08*	*1.14 ± 0.14*
Acat2	*1.0 ± 0.21*	*0.95 ± 0.05*	*Fibrosis*		
ApoB	*1.0 ± 0.18*	*0.98 ± 0.05*	Col1A1	*1.0 ± 0.06*	*1.10 ± 0.12*
Cyp7a1	*1.0 ± 0.26*	*0.89 ± 0.06*	Col3A1	*1.0 ± 0.06*	*1.01 ± 0.09*
Hmgcr	*1.0 ± 0.08*	*1.05 ± 0.07*	Fn1	*1.0 ± 0.04*	*1.07 ± 0.14*
Ldlr	*1.0 ± 0.25*	*0.81 ± 0.05*	Timp1	*1.0 ± 0.24*	*0.89 ± 0.15*
Srb1	*1.0 ± 0.52*	*0.37 ± 0.02*			
Vldr1	*1.0 ± 0.20*	*1.05 ± 0.09*			
*Nitrogen and fatty acid metabolism*					
Cps1	*1.0 ± 0.09*	*1.17 ± 0.08*			
Cpt1a	*1.0 ± 0.06*	*0.98 ± 0.06*			
Acox1	*1.0 ± 0.05*	*1.05 ± 0.02*			
Acaca	*1.0 ± 0.17*	*1.01 ± 0.19*			
Scd1	*1.0 ± 0.31*	*1.91 ± 0.30*			
Adipoq	*1.0 ± 0.58*	*0.99 ± 0.07*			
Adipor	*1.0 ± 0.05*	*0.93 ± 0.02*			
Ffar1	*1.0 ± 0.11*	*1.11 ± 0.06*			

The mice were fed diets containing different protein sources for 13 weeks. Data are mean ± SEM (*n* = 10–12)

^*^Indicate significant difference (p<0.05) between dietary treatments.

## Discussion

The main purpose of this study was to evaluate the effect of different protein sources on atherosclerotic development using female apoE^−/−^ mice fed a Western-type diet. The protein sources were chicken or cod and scallop (1:1 on nitrogen basis). The reason for combining cod and scallop was to create a lean marine protein source high in taurine and glycine. Chicken was chosen because it is considered a healthy and lean protein source of terrestrial origin [26]. Mice fed the cod-scallop diet for 13 weeks had significantly reduced aorta atherosclerotic plaque burden compared to chicken fed mice.

Diets rich in seafood are generally recommended to humans due to the triglyceride lowering and anti-inflammatory effects of EPA and DHA [27]. However, in the present study, the serum concentration of triglycerides did not differ significantly between the two groups. A triglyceride lowering effect of EPA and DHA has mainly been shown at pharmaceutical doses and is not commonly evident at nutritional levels [28] as given in the present study. When the cholesterol content in the two diets was analyzed, it was found that the chicken diet contained 0.20 % cholesterol whereas the cod-scallop diet contained 0.19 % cholesterol, but this small difference is not sufficient to cause a reduction in aorta atherosclerosis *per ce*. Taurine is known to reduce cholesterol levels in blood by increased cholesterol clearance from blood circulation, bioconversion of cholesterol to bile acid in liver and excretion of cholesterol and bile acids from the intestine [29]. Hence, the higher dietary level of taurine in the cod-scallop diet was expected to contribute to a reduced level of circulating cholesterol. Indeed, a reduced level of serum LDL cholesterol was observed in the cod-scallop fed mice compared to the chicken fed mice, whereas we did not find a significant reduction of serum total cholesterol in the cod-scallop fed mice compared to the chicken fed mice.

The mice fed chicken protein gained more weight than mice fed cod-scallop protein and body weight, visceral white adipose tissue weight and liver lipid content was higher in chicken fed mice compared to cod-scallop fed mice. A positive correlation between satiety and blood taurine concentrations in humans has been reported [30], and it has been demonstrated that taurine supplementation of male mice affected energy intake and prevented a high-fat diet induced obesity [31]. In this study, however, the feed consumption was equal in the two groups suggesting that metabolism was affected differently. In a recent study by Tastesen et al. [17], it was shown that dietary taurine and glycine correlated inversely with body fat mass. The mechanisms, however, remain unknown. Choice of dietary protein source has been documented to affect energy metabolism and to be associated with diet-induced obesity in rats [32, 33]. Rats fed fish protein hydrolysates have been shown to have elevated plasma bile acids and reduced visceral adipose tissue relative to a casein fed control group [32, 33]. The plasma concentrations of bile acids were not measured in this study, hence the exact mechanisms behind the weight gain difference, were not elucidated.

Furthermore, the cod-scallop fed mice had lower concentration of leptin compared to chicken fed mice. This is most probably due to the lower content of adipose tissue, which is the primary site of leptin production. Leptin is a key hormone in regulation of food intake and energy expenditure [34]. Lately it has also been demonstrated to play a direct role in almost every step in the development of atherosclerotic plaques [35]. It has previously been reported for both humans and rodents that obesity is associated with leptin resistance and that obese individuals have elevated leptin levels without any down-regulation of food intake [36]. Leptin is also indicated to function as a proinflammatory adipokine, and to further investigate the effect on the inflammatory response, we analyzed both circulating levels and hepatic expression of several inflammatory markers. Expression of the *Vcam1* gene encoding the cellular adhesion molecule VCAM-1 was lower in the cod-scallop fed mice. This protein mediates firm adhesion of leukocytes that then migrate into the sub-endothelium. The *Vcam1* gene is normally up regulated by hyperlipidemia and by inflammatory cytokines [37], proposing a potentially higher inflammatory condition in the chicken fed mice. However, hepatic expressions of inflammatory marker genes such as *Ccl2*, *Il1b*, and *Icam1* were not influenced and serum levels of inflammatory proteins were unaltered between the two groups. The level of atherosclerotic plaque formation observed in this study represents levels found in asymptomatic, apparently healthy humans [38] and not surprisingly the serum levels of cytokines and chemokines are unaffected by the dietary intervention in this model.

It is well accepted that oxidative stress may accelerate the development of atherosclerosis [39] with subsequent up-regulation of cellular antioxidants such as the protein PON2 [40]. In the cod-scallop fed mice the expression of the *Pon2* gene was lower compared to chicken fed mice, suggesting lower oxidative stress, which may be a possible explanation for the reduced atherogenesis. Taurine is known to possess antioxidative capacity [41] and may have attributed to the lower oxidative stress in the cod-scallop fed group.

In the present study, the dietary concentrations of n-3 PUFAs were higher, and of SFA and MUFA were lower in the cod-scallop diet as compared to in the chicken diet. Compared to the chicken fed mice, the mice fed cod-scallop diet had similar liver levels of SFA, lower levels of MUFA and higher levels of EPA, DPA and DHA, and further a correspondingly lower n-6/n-3 ratio. An antiatherosclerotic effect of marine n-3 PUFA have previously been observed both in the apoE^−/−^ mouse model [21] and in the Ldlr^−/−^ mouse model [42–45]. As previously mentioned, the amounts of n-3 PUFA given in the present study were lower compared to the previous studies. However, we cannot exclude the possibility that the dietary fatty acid composition contributed to the observed effects in the present study.

In a clinical human setting, atherosclerosis leads to hemodynamic stress often followed by a compensatory hypertrophic growth of the heart and ultimately plaque rupture and ischemic heart disease. To search for detectable differences in cardiac gene expression of markers of heart function and homeostasis, fibrosis, apoptosis and angiogenesis, RNA was isolated from cardiac apices from the mice. No differences were found between mice fed chicken and mice fed cod-scallop in any of the tested genes, indicating that the diets did not affect the heart. This was supported by the lack of hypertrophic growth of the cardiac muscle. There is however little evidence indicating that the heart would be affected considering the low levels of dietary cholesterol given to the mice and the low atherosclerotic burden observed in the mice, and substantial effects on the heart was not expected.

## Conclusions

This study has demonstrated a beneficial metabolic effect in the mice fed cod-scallop compared to the mice fed chicken, as aorta atherosclerotic burden, body weight, visceral adipose tissue, serum glucose and leptin levels, all were reduced. The results warrant further investigations to elucidate in detail the molecular mechanisms underlying the observed effects.

## Additional file

Additional file 1: Table S1. Predesigned TaqMan® Gene Expression assays used. (DOCX 16 kb)
