# Supplementary material for: Dietary intake of cod and scallop reduces atherosclerotic burden in female apolipoprotein E-deficient mice fed a Western-type high fat diet for 13 weeks
Source: Nutr Metab (Lond). 2016 Feb 2;13:8. doi: 10.1186/s12986-016-0068-z (PMC4735963; doi:10.1186/s12986-016-0068-z)
Supplement: Additional file 1: Table S1. — Predesigned TaqMan® Gene Expression assays used. (DOCX 16 kb) [file 12986_2016_68_MOESM1_ESM.docx]

# Additional file 1

Table S1 Predesigned TaqMan® Gene Expression assays used.

| **Abbreviation** | **Gene name** | **Assay #** |
| --- | --- | --- |
| *Abcg5* | ATP binding cassette, sub-family G member 5 | Mm00446241_m1 |
| *Abcg8* | ATP binding cassette, sub-family G member 8 | Mm00445980_m1 |
| *Acaca* | Acetyl-Coenzyme A carboxylase | Mm01304257_m1 |
| *Acat2* | Acetyl-Coenzyme A acetyltransferase 2 | Mm00782408_s1 |
| *Acox1* | Acyl-Coenzyme A oxidase 1 | Mm01246831_m1 |
| *Adipoq* | Adiponectin | Mm00456425_m1 |
| *Adipor* | Adiponectin receptor | Mm01291334_mH |
| *Agtr1a* | Angiotensin II receptor typre 1A | Mm01957722_s1 |
| *Apob* | Apolipoprotein B | Mm01545156_m1 |
| *Ankrd* | Ankyrin repeat domain-containing protein 1 | Mm00496512_m1 |
| *Bcl2* | B-cell lymphoma 2 | Mm00477631_m1 |
| *Casp3* | Caspase 2 | Mm01195085_m1 |
| *Ccl2* | Chemokine (C-C motif) ligand 2 | Mm00441242_m1 |
| *Col1a1* | Collagen type 1 alpha 1 | Mm00801666_g1 |
| *Col3a1* | Collagen type 3 alpha 1 | Mm01254476_m1 |
| *Cps1* | Casbamoyl-phosphate synthase 1 | Mm01256489_m1 |
| *Cpt1a* | Carnitine Palmitoyltransferase 1a | Mm01231183_m1 |
| *Cyp7a1* | Cytochrome P450 7A1 | Mm00484150_m1 |
| *Eif2bl2* | Eukaryotic translation initiation factor 2B, subunit 1 | Mm01199614_m1 |
| *Ffar1* | Free fatty acid receptor 1 | Mm00725193_m1 |
| *Flt1* | VEGF-receptor | Mm00438980_m1 |
| *Fn1* | Fibronectin 1 | Mm01256744_m1 |
| *Gpx1* | Glutathione peroxidase 1 | Mm00656767_g1 |
| *Gpx4* | Glutathione peroxidase 4 | Mm00515041_m1 |
| *Hmgcr* | 3-hydroxy-3-methyl-glutaryl-Coenzyme A reductase | Mm01282499_m1 |
| *Hprt* | Hypoxanthine-guanine phosphoribosyltransferase | Mm01545399_m1 |
| *Icam1* | Intercellular adhesion molecule 1 | Mm00516023_m1 |
| *Il1b* | Interleukin 1beta | Mm00434228_m1 |
| *Ldlr* | LDL-receptor | Mm01177349_m1 |
| *Myh6* | Myosin heavy chain 6 | Mm00440359_m1 |
| *Myh7* | Myosin heavy chain 7 | Mm01319006_g1 |
| *Nfe212/Nrf2* | Nuclear factor erythroid 2-related factor | Mm00477784_m1 |
| *Nppa* | Natriuretic peptide precursor A | Mm01255747_g1 |
| *Nppb* | Natriuretic peptide precursor B | Mm01255770_g1 |
| *Pon2* | Paroxynase 2 | Mm00447159_m1 |
| *Scarb1* | Scavenger receptor class B member 1 | Mm00450234_m1 |
| *Scd1* | Stearoyl-Coenzyme A desaturase-1 | Mm00772290_m1 |
| *Sod1* | Superoxid dismutase 1 | Mm01344233_g1 |
| *Sod2* | Superoxid dismutase 2 | Mm01313000_m1 |
| *Timp1* | Tissue inhibitor of matrix metalloprotease-1 | Mm00441818_m1 |
| *Tbp* | TATA-Box Binding Protein | Mm00446971_m1 |
| *Ucp2* | Uncoupling protein 2 | Mm00627599_m1 |
| *Vcam1* | Vascular adhesion molecule 1 | Mm01320970_m1 |
| *Vegfa* | Vascular endothelial growth factor A | Mm01281449_m1 |
| *Vegfb* | Vascular endothelial growth factor B | Mm00442102_m1 |
| *Vldlr* | Very low density lipoprotein receptor | Mm00443298_m1 |
